# Improving readiness for recruitment through simulated trial activation: the Adjuvant Steroids in Adults with Pandemic influenza (ASAP) trial

**DOI:** 10.1186/s13063-017-2290-z

**Published:** 2017-11-16

**Authors:** Wei Shen Lim, Garry Meakin, Clare Brittain, Thomas Bewick, Lelia Duley

**Affiliations:** 1 Respiratory Medicine, Nottingham University Hospitals NHS Trust, City Hospital Campus, Nottingham, NG5 1 PB England UK; 2 Nottingham Clinical Trials Unit, University of Nottingham, Queen’s Medical Centre, Nottingham, NG7 2UH England UK; 3 Respiratory Medicine, Derby Teaching Hospitals NHS Foundation Trust, Uttoxeter Rd, Derby, DE22 3NE England UK

**Keywords:** Pandemic, Influenza, Clinical trial, Simulation, Hibernation, Corticosteroids, Delay, Trial delivery

## Abstract

**Background:**

Research in public health emergencies requires trials to be set up in readiness for activation at short notice and in anticipation of limited timelines for patient recruitment. We conducted a simulated activation of a hibernating pandemic influenza clinical trial in order to test trial processes and to determine the value of such simulation in maintaining trial readiness.

**Methods:**

The simulation involved the Nottingham Clinical Trials Unit, one participating hospital, one manufacturing unit and the Investigational Medicinal Product (IMP) supplier. During the exercise, from 15 September 2015 to 2 December 2015, clinical staff at the participating site completed the trial training package, a volunteer acting as a patient was recruited to the study, ‘dummy’ IMP was prescribed and follow-up completed.

**Results:**

Successful activation of the hibernating trial with patient recruitment within 4 weeks of ‘arousal’ as planned was demonstrated. A need for greater resilience in anticipation of staff absenteeism was identified, particularly in relation to key trial procedures where the potential for delay is high. A specific issue relating to the IMP Stock Control System was highlighted as a potential source of error that could compromise the randomisation sequence. The simulation exercise was well received by site investigators and increased their confidence in being able to meet the likely demands of the trial when activated. The estimated cost of the exercise was £1995; 90% of this being staff costs.

**Conclusions:**

Simulated activation is useful as a means to test, and prepare for, the rapid activation of ‘hibernating’ research studies. Whether simulation exercises can also help reduce waste in complex clinical trial research deserves further exploration.

**Trial registration:**

EudraCT Number 2013-001051-12, ISRCTN72331452. Registered on 6 March 2013.

## Background

There is growing recognition that preparedness for public health emergencies, such as pandemics, earthquakes or terrorist attacks, should include the development and set-up of appropriate research ready for rapid activation [1–4]. As the window of opportunity for research during such emergencies is often limited, clinical trials set up to respond to these emergencies are particularly vulnerable to delays in trial processes.

In multicentre trials, once a site is set up and ready to start, a common inefficiency is delay in recruiting the first participant; this, in turn, is associated with poor overall trial accrual [5]. Careful trial design and set-up by experienced clinical trial units and investigators can mitigate against delays arising due to deficiencies in trial processes. Nevertheless, differing local situations at sites, or unique trial-specific requirements, may create hurdles that are not easily anticipated.

Increasingly, internal pilot studies form an integral part of large multicentre trials [6]. One of the roles of pilot studies is the identification and, if possible, correction of deficiencies or obstacles in trial processes that may adversely affect recruitment [7]. However, in certain circumstances, such as in trials to be conducted during public health emergencies or outbreaks of disease, pilot studies may not be appropriate, or even possible [1, 8].

The Adjuvant Steroids in Adults with Pandemic influenza (ASAP) trial has been set up to determine if the early use of low-dose corticosteroids in adults hospitalised with pandemic influenza is beneficial [9]. This trial is part of the UK NIHR pandemic research portfolio. In order for the results of the trial to inform and influence clinical management and health policy within the same pandemic, the trial will need to complete recruitment within the first few weeks of the pandemic, with primary outcome results reported as soon as possible thereafter. Specifically, the ASAP trial will recruit the first participant within 28 days of activation, and complete recruitment of 2200 participants at approximately 40 sites in the UK within the first pandemic wave; a duration of approximately 6 weeks. Minimising obstacles that might arise during recruitment and require ‘time-costly’ solutions is critical to the success of the trial. There will be no time to conduct and evaluate a pilot or feasibility study.

Simulation exercises are a recognised means for developing and improving operational readiness within clinical practice [10, 11]. We conducted a simulated activation of the ASAP trial in order to (1) test trial processes and identify potential problems associated with trial delivery, (2) enhance training of site investigators and (3) to determine the value of such simulation in maintaining trial readiness.

## Methods

### Participating units and staff

The simulation exercise was conducted from 15 September 2015 to 2 December 2015 involving the Nottingham Clinical Trials Unit (NCTU), Derby Teaching Hospitals NHS Foundation Trust (DTHFT, a participating research site), Rosemont Pharmaceuticals (supplier of the Investigational Medicinal Product (IMP) and placebo product) and Nottingham Pharmacy Production Unit (one of three manufacturing units supporting the trial). Approvals for the conduct of the ASAP trial at DTHFT were in place from 11 July 2014, after which the trial was placed in ‘hibernation’. Research personnel involved at DTHFT included the principal investigator, pharmacist and research nurses. A junior physician with no prior knowledge of the ASAP trial agreed to volunteer to participate in the exercise as a ‘patient’; the volunteer gave informed consent towards their participation. The timing of the simulation exercise was pre-determined with the cooperation of DTHFT site investigators and involved testing all relevant trial processes from the issue of a trial activation alert through to follow-up of 30-day post-charge secondary outcomes.

### Simulated activation process

**Fig. 1:**
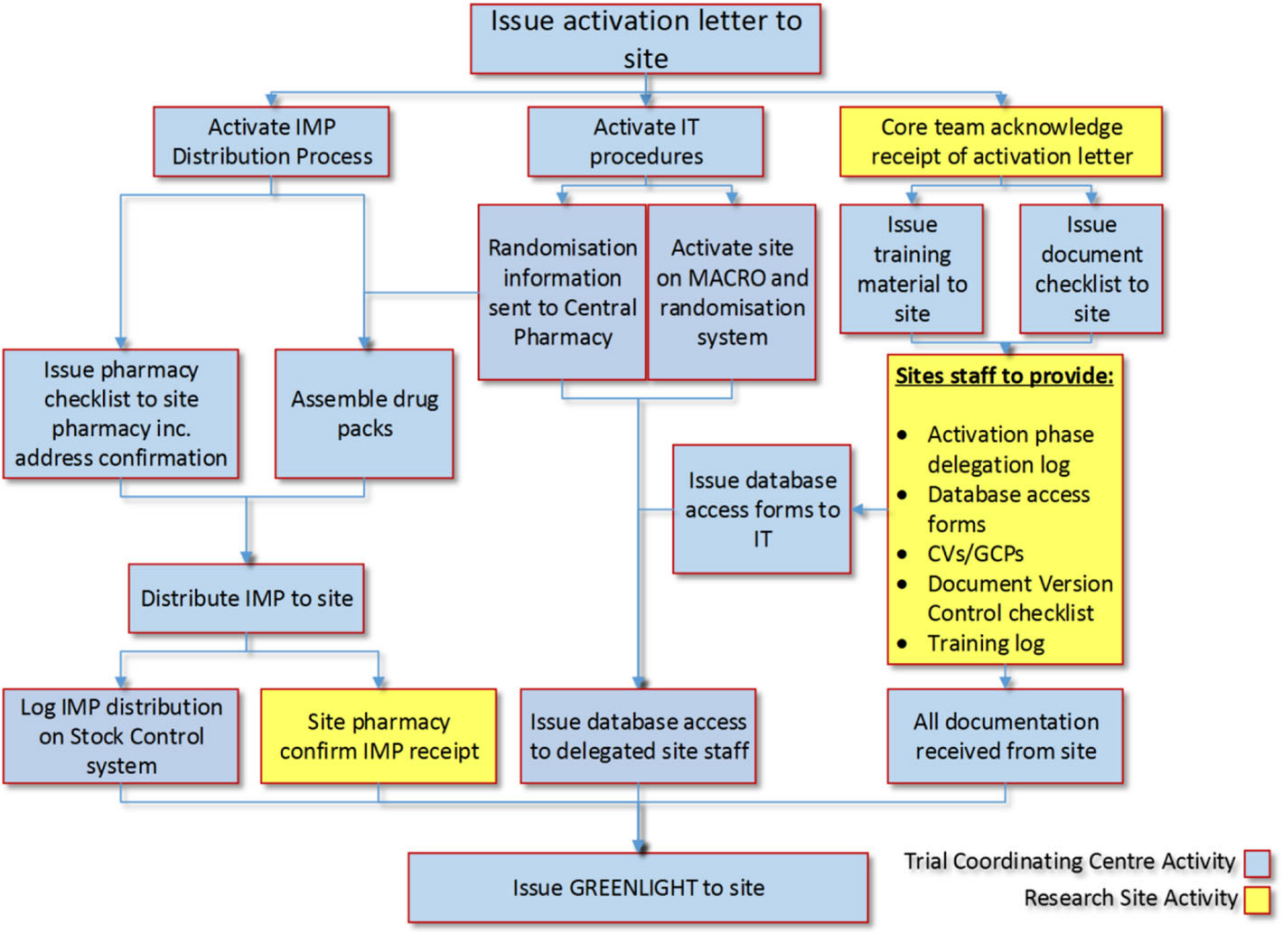
Simulated activation flowchart

The trial was activated by the NCTU on 15 September 2015. Figure 1 outlines that range of activities performed following activation. The volunteer ‘patient’ was asked to arrive at the Medical Admissions Unit (MAU) at a time of their choosing 1 month post activation (15 October 2015). Five members of the acute medical team from the Medical Admissions Unit at DTHFT were asked to complete the trial specific on-line training within 4 weeks of activation (the ASAP trial is set up to be able to recruit no later than 4 weeks from activation). The IMP/placebo product was delivered to DTHFT via usual distribution channels as planned in the protocol and local operating procedures for the ASAP trial; sterile water was used as the trial treatment for the simulated activation.

On the day of recruitment, the local principal investigator (PI) posed as the volunteer patient’s relative, and recorded the time required for trial processes involving the patient, such as time required to take consent and time from consent to administration of the IMP; only the PI from the research team was aware of this recording.

The simulation included ‘live’ prescription for the IMP on the trust’s electronic prescribing system through to administration of the IMP to the volunteer patient but did not include taking of the IMP by the volunteer patient. The research team at DTHFT entered dummy data into the Case Report Form. All follow-up procedures were performed according to the trial protocol including sending of the postal questionnaire to the patient 30 days after hospital discharge.

### Evaluation

A post-simulation meeting with the DTHFT research team was convened 3 weeks after ‘recruitment’ to assess acceptability of the exercise and to share lessons learnt.

Cost expenditure associated with the simulation exercise were calculated using the Nottingham University Hospitals Research and Innovation Non-commercial Costing Template (Issued 8 November 2016). The costing for staff time was based on mid-point of Agenda for Change NHS banding. All costs were reviewed and approved by relevant third parties.

## Results

### Conduct of simulated activation

The trial was activated on 15 September 2015 and IMP requested from the manufacturer on the same day. The IMP was delivered to the manufacturing unit 3 days later (18 September 2015). Following quality control (QC) checks by the manufacturing unit and subsequent qualified person (QP) release, IMP was received by the participating site pharmacy on 13 October 2015 (28 days from activation) and placed in a designated secure area in the MAU according to local trial plans.

Patient recruitment took place on 15 October 2015 (30 days from activation). The total time from research nurses being alerted of the volunteer patient’s arrival at the MAU to the IMP being dispensed was 23 min (Table 1). The patient was discharged from hospital on the day of recruitment and given instructions regarding the continuation of IMP at home; no IMP was actually given to the patient to take home. A postal questionnaire was sent to the patient on 16 November 2015 with a response returned on 2 December 2015 (47 days from hospital ‘discharge’).

**Table 1:** Improvements following simulated trial activation

Activity	Key observations	Benefit from simulation
Communication	1. Prompt responses across all parties 2. Incorrect contact details identified 3. Concerns identified regarding resilience of cover in the event of absenteeism of key personnel associated with trial processes	1. Improved levels of communication between trial partners 2. Highlighted need for building greater trial resilience at all levels – key personnel have been identified and asked to organise appropriate pandemic cross-cover; e.g. 2 ‘deputy’ chief investigators have been ‘appointed’
Documentation	4. Outdated documents identified 5. Training material well received by clinical and research staff	3. Improved quality control oversight of trial documentation – all trial documents are now available from the ASAP trial website to enable rapid updating and dissemination in a pandemic situation; QC checks at pre-determined time-points to ensure version control of all trial documents has been added as a critical trial procedure for the Coordinating Centre 4. Confirmation of adequacy of trial-specific training material
Pharmacy	6. IMP storage arrangements questioned by QP 7. Stock Control System requirements a risk to randomisation sequence 8. Time frames for delivery of IMP not stipulated in ‘Agreements’	5. Clarification and confirmation of IMP storage standards – local pharmacy now required to confirm receipt of distribution carton pre-packed with 6 IMP packs, without opening the carton and potentially disrupting the randomisation sequence 6. Stock Control System amended to protect randomisation procedure 7. IMP-related processes revised
Data collection/ database	9. Electronic CRF worked well 10. Database functioned well	8. Verification of IT and database processes
Site staff and recruitment	11. Good engagement 12. Good knowledge of the trial	9. Increased confidence of site investigators 10. Verification of required research infrastructure 11. Confirmation of readiness for rapid activation

*ASAP* The Adjuvant Steroids in Adults with Pandemic influenza trial, *CRF* Case Report Form, *IMP* Investigational Medicinal Product, *IT* information technology, *QC* quality control, *QP* qualified person

### Feedback from site staff

Clinical staff who undertook the trial-specific training package reported that the format and content of the training material was clear, precise and relevant to their role in the trial. Site investigators reported that sufficient information was provided to them for the conduct of the trial safely and comprehensively. They felt the simulated activation had increased their confidence regarding trial delivery and were supportive of such simulations being conducted at other sites as well.

### Issues identified

Two important issues relating to the distribution and handling of IMPs were identified. Firstly, a delay was encountered in the QC processes following receipt of the IMP at the manufacturing unit. This was due to the person qualified to conduct the QC – the qualified person (QP) – being away without suitable cover arrangements in place. On return, the QP subsequently raised concerns that the amber-coloured bottle used to store the IMP was not sufficient to protect the active compound from light degradation once the bottle was removed from its box and placed in the transparent trial packaging. Clarification on this issue had to be obtained from the manufacturer to the satisfaction of the QP.

**Fig. 2:**
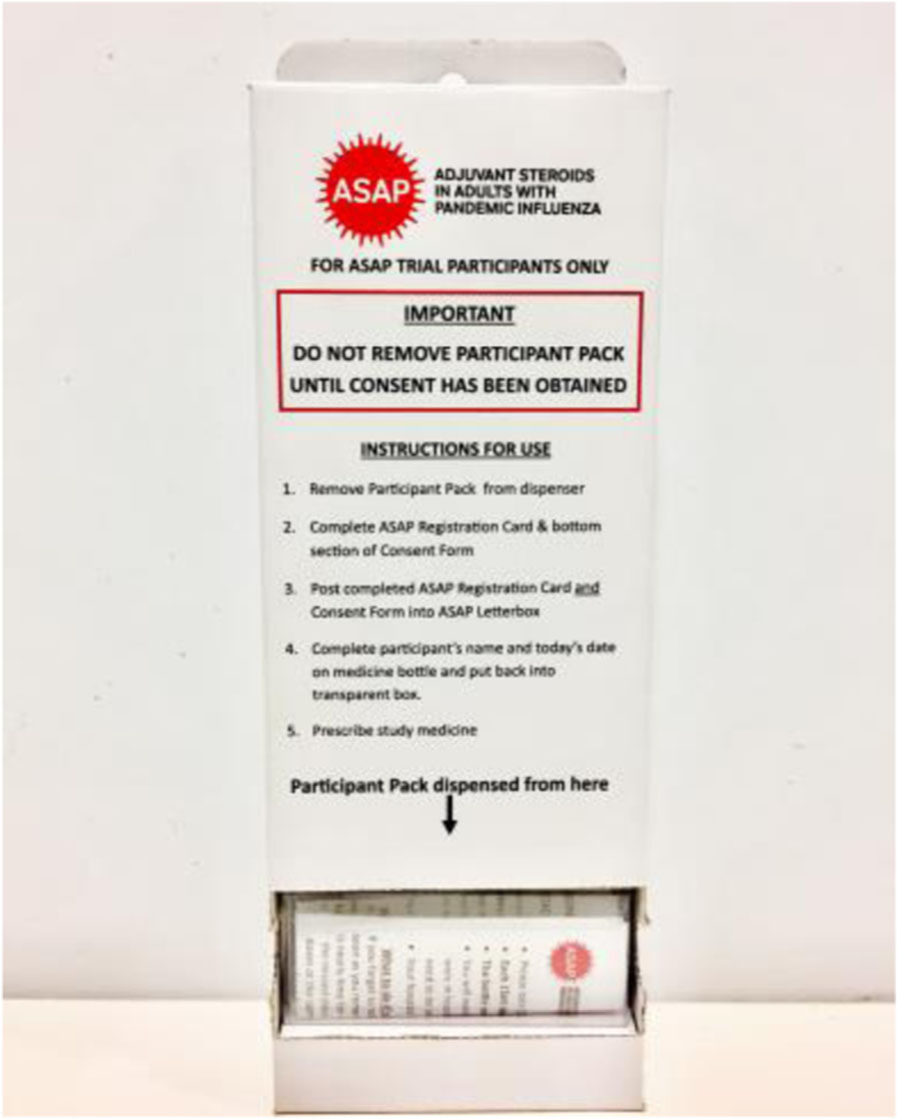
Investigational Medicinal Product (IMP) distribution carton

Secondly, site pharmacy staff reported that the Stock Control System required the logging of receipt of each individual IMP pack; this entailed removing each pack from the distribution carton which contained six IMP packs within (see Fig. 2). The distribution carton had been specially designed for this trial to minimise potential delays in obtaining trial IMP/placebo within the context of high clinical demands during a pandemic. The sequence of packs within the distribution carton was an integral component of the randomisation sequence. The Stock Control System requirements, therefore, had the potential to compromise the randomisation sequence.

Other minor improvements relating to communication channels, trial documentation, trial database set up and verification of processes were identified (Table 1).

### Cost of simulation exercise

The overall estimated cost of the simulated activation was £1995. Of this, one-off set-up costs incurred by the Coordinating Centre were £1018 (Table 2); these costs would have been invariable regardless of the number of sites activated. Overall, 90% of costs related to staff costs (£1796).

**Table 2:** Costs of simulated trial activation

Action	Item/staff	Time (mins)	Cost
A. One-off costs at Coordinating Centre			
Internal planning meeting(s)	Multiple staff	360	£690.60
Mock activation planning meetings	Multiple staff	120	£206.76
Development of simulation documents	Trial manager	120	£39.88
QC of website	QA manager	120	£44.02
Review training material	Trial manager	60	£19.94
Activation of IT systems	Database programmer	60	£17.72
		Sub-total	£1018.92
B. Costs of site simulation Coordinating Centre			
Planning meeting with site	Multiple staff	60	£115.10
Distribution of documents	Trial administration	40	£8.79
Activation of IT systems	Multiple staff	120	£35.44
Obtain and despatch IMP	Multiple staff	50	£14.73
Issue green light	Trial manager	10	£3.32
Follow-up questionnaire	Trial administration	20	£3.85
Non-staff expenses	Cost of IMP/packaging		£132.50
	IMP delivery		£60.00
	Trial team transport		£6.00
		Sub-total	£379.73
Research site			
Planning meetings	Multiple staff	60	£107.57
Complete online training	Multiple staff	20	£156.16
Complete documents/forms	Multiple staff	25	£183.13
Recruit patient to study	Research nurse	5	£12.64
		Sub-total	£459.50
Central pharmacy			
Planning meetings	Specialist pharmacist	60	£22.89
Receive and dispatch IMP	Specialist pharmacist	5	£47.70
QP release	QP	60	£65.72
		Sub-total	£136.31
		Total	£1994.46

*IMP* Investigational Medicinal Product *IT* information technology, *QA* quality assurance *QC* quality control*, QP* qualified person

## Discussion

This activation exercise confirmed that the ASAP trial is in a state of readiness and can be ‘aroused’ from its current hibernation phase to active recruitment within 4 weeks of activation, as planned [9]. Important practical issues that had the potential to delay the time from trial activation to first patient recruitment were identified and addressed.

A need for greater trial resilience was identified in relation to absenteeism of key personnel important to trial delivery. During a pandemic, it is estimated that up to 50% of staff may be absent at some point due to illness, or the need to care for family members who fall ill [12]. Trial Coordinating Centres need to have suitable pandemic plans in place, including approved cover arrangements for the chief investigator and other key personnel. Considerations of resilience extend to the membership and functioning of the Trial Steering Committee, the Data Monitoring Committee, and to elements potentially outside the immediate influence of the trial team, such as the IMP/placebo product supplier and QPs. Although the trial team had already developed what were thought to be appropriate pandemic resilience plans, the simulation exercise revealed further areas for attention.

In the design of the ASAP trial, it was recognised that conducting a trial during a pandemic would be highly challenging due to demands on clinical staff and healthcare services. Specifically in relation to randomisation, it was determined that a web-based or telephone-based process which required a clinician to contact the trial team for the randomisation schedule would be too time-consuming and would deter clinicians recruiting potential participants. Therefore, it was designed that IMP would be pre-packed in distribution cartons according to a computer-generated random sequence. The distributions cartons would allow for easy storage at hospital admission points and crucially, would allow clinicians to simply take the next IMP pack from the distribution carton as part of the randomisation process. However, the well-established Stock Control System used by the CTU and which was designed primarily for a web-based randomisation process, required the local pharmacy to remove individual IMP packs from the distribution cartons in order to confirm receipt of IMP; with the potential to disturb the randomisation sequence. That this potential problem was not identified during trial set-up is likely due to the mismatch between ‘usual’ versus ‘unique’ trial-specific processes.

The costs for the simulation exercise were relatively modest compared to the overall costs of the trial. We were unable to reliably estimate likely cost savings arising from improvements made to trial procedures as a result of the simulation exercise. Anticipated cost savings are dependent on the ‘cost’ of slow trial accrual. In the case of pandemic and public health emergency trials, slow accrual may result in failure to complete a trial with enormous attendant costs in economic and human terms [13].

The single site design of the simulation exercise is a study limitation. Potentially, involvement of different sites might identify different areas for improvement. However, the majority of issues identified during the simulation exercise were relevant to the entire trial and were not site-specific. Hence, repeated simulation exercises involving multiple sites within a single trial may be associated with decreasing amounts of learning and be inappropriate. On the other hand, some of the major benefits of a simulation exercise derive simply from enabling local sites to rehearse their trial processes; hence identifying outdated local processes or new obstacles to trial delivery which may have arisen during the hibernation period. These benefits are not manifest as improvements to the trial but as the equally important requirement to maintain local site trial readiness and interest. Performing simulation exercises across all sites, whether in clusters or in totality, will require resources which need to be recognised by all parties, including trial funders. Inevitably, the simulation exercise included elements that were artificial, such as the involvement of a junior physician as a ‘simulated patient’. Future improvements to the simulation design would include employing professional actors as ‘patients’, as is common in simulated medical training exercises. In addition, a large number of ‘patients’ could be asked to present at the same time, thus simulating the crowd pressures that would exist during a pandemic. These improvements would increase the veracity of the simulation exercise but would also be associated with higher costs. Ultimately, awareness of those areas where simulation biases towards improved performance, or artificiality, enables avoidance of unjustified conclusions.

### Implications

A major challenge in the conduct of studies during public health emergencies is the requirement to rapidly recruit sufficient participants before the end of the emergency; there is no option to extend the recruitment period because of obstacles encountered during trial delivery or slow accrual [14]. During the 2009 influenza pandemic, a phase III trial of corticosteroids in critically ill patients was initiated 2 to 3 weeks following the peak of the pandemic and only managed to recruit 26 participants before the end of the pandemic; the required sample size was 438 participants [15]. Similarly, during the 2014 Ebola outbreak in east Africa, at least three clinical trials were initiated without managing to recruit the required number of participants before the end of the outbreak [13, 16]. These uncompleted trials leave important clinical questions unanswered. Simulation exercises allow potential procedural delays to trial delivery to be identified and tackled prior to trial activation. This is a major benefit particularly for public health emergency trials.

Simulation exercises may also be useful for trials with complex or unusual trial processes. The importance of trial systems and procedures for efficient trial conduct is well recognised [17]. In most circumstances, an experienced clinical trial team should be able to pre-emptively identify all relevant potential hurdles to trial delivery and address these prior to trial activation. However, when trial processes deviate from standard patterns, it can be more difficult to anticipate potential problems.

## Conclusions

Simulation exercises to test the adequacy of rapid ‘arousal’ procedures and trial delivery processes should be considered as a component of the set-up of ‘hibernating’ research studies. Whether simulation exercises can also help reduce waste in complex clinical trial research deserves further exploration [18].

## Abbreviations

ASAP: Adjuvant Steroids in Adults with Pandemic Influenza
CTU: Clinical Trials Unit
DTHFT: Derby Teaching Hospitals NHS Foundation Trust
IMP: Investigational Medicinal Product
MAU: Medical Admissions Unit
NCTU: Nottingham Clinical Trials Unit
QC: Quality control
QP: Qualified person
